# Research progress on the mechanism of panax notoginseng saponin in the treatment of atherosclerosis

**DOI:** 10.3389/fcvm.2026.1659763

**Published:** 2026-02-05

**Authors:** Long Xiong, Dongli Liu, Jiao Cheng, Tingfu Yin

**Affiliations:** Department of Geriatrics, Zhenxiong County Hospital of Traditional Chinese Medicine, Zhaotong, Yunnan, China

**Keywords:** anti-inflammation, anti-oxidant stress, atherosclerosis, panax notoginseng, panax notoginseng saponin, pharmacological effect

## Abstract

With the increasing incidence and prevalence of atherosclerosis (AS), it has become a major global public health concern. While interventional surgery and Western medicines are effective in treating AS, their adverse effects are noteworthy. Traditional Chinese medicine offers the advantage of low side effects and significant therapeutic effects, making its active ingredients potential candidates for new medicine development. Panax notoginseng saponin (PNS), a compound derived from the natural drug panax notoginseng, has shown significant efficacy in AS treatment by targeting six main aspects: anti-inflammation, regulation of lipid metabolism, anti-oxidative stress, inhibition of vascular smooth muscle cell proliferation and migration, inhibition of angiogenesis, and regulation of autophagy. This study reviews the pharmacological mechanisms of PNS in AS treatment, as well as its biological properties, *in vivo* metabolism, synthesis, and regulation, providing insights for future research.

## 1 Introduction

Atherosclerosis (AS) is a long-lasting chronic inflammatory disease of large and medium-sized arteries (1); it can further develop into a wide range of cardiovascular diseases (CVD) such as coronary heart disease and cerebral infarction (2). CVD, the incidence of which increases gradually with age, are major diseases that seriously jeopardize human health and life and are the leading cause of death and reduced quality of life worldwide (3, 4). As a result, there is an urgent need to ramp up research on therapeutic drugs for AS. The pathology of AS is characterized by impaired endothelial lipid deposition or inflammatory response in the arteries, platelet aggregation in the endothelium, foam cell formation, and formation of atherosclerotic plaques, leading to lumen narrowing and wall thickening (5). In addition, the plaque formative process includes intra-plaque bleeding, plaque bursting, and calcification, which leads to the formation of blood clots, arterial vascular occlusion, and ischemia or necrosis of tissues or organs (6), which in severe cases can lead to acute myocardial infarction, cerebral infarction and other diseases. Nowadays, AS has now become a major public health problem of global concern.

With the development of medical technology, the efficacy of interventional and revascularization procedures for the treatment of atherosclerotic disease is remarkable, but they can greatly increase the economic burden on society and patients. The main medication drugs for AS include statins and aspirin, which generally require lifelong use. Numerous studies have proven drug resistance and adverse effects of statins and aspirin when taken for long periods (7–9). Statins and proprotein convertase subtilisin/kexin 9 (PCSK 9) inhibitors decrease low-density lipoprotein (LDL)-cholesterol to low levels, but do not eliminate the cardiovascular risk of other factors contributing to AS or the atherosclerotic cardiovascular disease pathway (10). As a result, the search for new therapeutic approaches and natural drugs in treating AS has received much attention. Traditional Chinese medicine (TCM) has significant efficacy in treating CVD, with enjoys strengths multi-targeting, low cost, and less negative effects, and is becoming increasingly popular worldwide (11); it treats CVD by regulating lipid metabolism, oxidative stress, inflammatory response, and other mechanisms (12).

Panax notoginseng saponin (PNS) is the main component of panax notoginseng. Modern studies have proved that PNS have various pharmacologic effects, such as adjusting lipid metabolism (13), anti-inflammatory (14), anti-oxidative stress (15), anti-cancer and regulating intestinal microbiota (16). PNS is widely used in treating AS, CVD, diabetes, cancer and other diseases (17, 18). This paper describes six aspects of the role of PNS on AS and its mechanism from anti-inflammation, regulation of lipid metabolism, anti-oxidative stress, inhibition of vascular smooth muscle cells (VSMCs) proliferation and migration, inhibition of angiogenesis and regulation of autophagy. In addition, we present PNS sources, metabolism, synthesis and regulation. Our review will provide a basis for the clinical value of PNS.

## 2 Biological characteristics of panax notoginseng

### 2.1 The name of panax notoginseng

Panax notoginseng (Burk.) F. H. Chen has been used for more than 600 years and is one of the herbs widely used in TCM. Panax notoginseng belongs to the pentacarpaceae family and was first recorded in the “Compendium of Materia Medica (1785)” written by Shizhen Li, abbreviated as tian qi, jin buhuan, which can stop bleeding, disperse blood and relieve pain. It is widely used in treating CVD, such as AS, ischemic brain injury, and occlusive vasculitis.

### 2.2 The primary active component of panax notoginseng saponin

**Figure 1:**
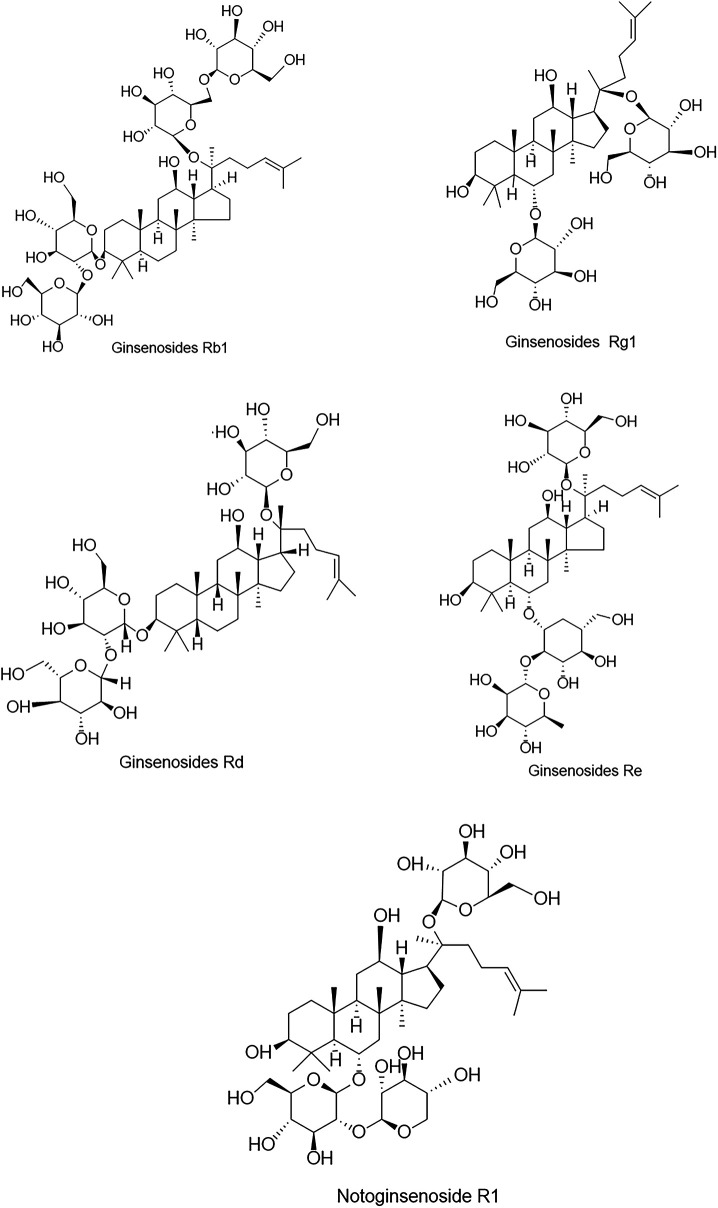
Chemical structures of the five major components in PNS.

Panax notoginseng containing about 200 compounds, including saponins, flavonoids, cyclic peptides, sterols, and amino acids, etc., of which PNS is the major activity component of panax notoginseng (19). PNS are predominantly dammarane triterpenes with 20 (S)-protobirdiol or 20 (S)-protobirditriol aglycon moieties and contain about 30 kinds of saponins, among them, ginsenosides Rb1 (Rb1), ginsenosides Rg1 (Rg1), ginsenosides Rd (Rd), ginsenosides Re (Re) and notoginsenoside R1 (NGR1) were the most abundant (20), and their chemical structures (Figure 1). Drugs with PNS as the main ingredient include xueshuantong injection, xuesaitong injection, etc., which are widely used in treating acute myocardial infarction, angina pectoris, and cerebral infarction (21–25).

### 2.3 Biosynthesis and regulation of ginsenosides in Panax notoginseng saponin

The biosynthesis of ginsenosides involves three stages: formation of the ginsenoside skeleton, glycosome synthesis, and modification of the skeleton (26). The biosynthesis of ginsenosides involves the mevalonic acid (MVA) pathway in the cytoplasm and the methylerythritol phosphate (MEP), which are largely similar in Panax notoginseng (27). First, the terpenoid precursor isopentenyl diphosphate (IPP) and its isomer dimethylallyl diphosphate (DMAPP) are synthesized, then converted into geranyl pyrophosphate and farnesyl pyrophosphate (FPP). Two molecules of FPP combine via squalene synthase to form squalene (28). Squalene is converted by squalene epoxidase and dammarane diol synthase into 2,3-epoxysqualene, which undergoes hydroxylation by cytochrome P450 enzymes and glycosylation by uridine diphosphate-dependent glycosyltransferases to ultimately form ginsenosides (29). Transcription factors participate in the synthetic regulation of PNS by upregulating the expression levels of PnSS, PnSE, and PnDS, thereby increasing the levels of R1, Rg1, Re, Rb1, and Rd (30). PnNAC2 binds to PnSS, PnSE, and PnDS, activating their transcription to promote the synthesis of PNS (31).

### 2.4 The *in vivo* metabolism of panax notoginseng saponin

PNS exhibit poor absorption in human blood and have very low oral bioavailability. In recent years, increasing research has focused on the biotransformation of exogenous substances and drug metabolism mediated by the gut microbiota (32). Research indicates that glycosylation is the primary metabolic pathway for panaxosides through the gut microbiota, with the main metabolites being α-F1, protopanaxatriol (PPT), α-Rh 2, ginsenoside compound K (GCK), and protopanaxadiol (PPD) (33). Researchers employed UPLC-MS/MS to determine the concentrations of 28 PNS components in rat plasma and investigated the pharmacokinetics. Results indicated that Rb1, Rg1, R1, Re, and Rd were the primary plasma components detected after PNS administration. Following oral dosing, Rb1 and Rd accounted for 60% of total PNS exposure, while Rg1 and R1 accounted for only 0.7%. Re was not detected in plasma. The primary metabolites *in vivo* were PPD, PPT, and CK. PPD-type ginsenosides may be the primary pharmacologically active components in the body following oral administration. PPD-type glycosides such as Rb1 and Rd are first biochemically transformed to diglycosylated ginsenoside F2 and then to monoglycosylated GCK, and finally metabolized into deglycosylated ginsenoside PPD (34). The biotransformation pathways of PNS in gut microbiota-mediated metabolic processes include products of isomerization, hydration, dehydration, hydrogenation, dehydrogenation, oxidation, and acetylation reactions. The primary metabolites *in vivo* and *in vitro* are GCK and Rh2, with deglycosylation reactions being the main metabolic pathway (35).

## 3 Effect and mechanism of panax notoginseng saponin in atherosclerosis

Numerous research studies have identified hyperlipidemia, diabetes mellitus, hyperuricemia, homocysteinemia, smoking, and obesity as risk factors for AS. Recent investigations have shown that PNS, a natural drug, has potential in treating AS by modulating lipid metabolism and inflammation, inhibiting VSMCs proliferation and migration, and improving vascular endothelial cell injury (36–38). With significant efficacy and minimal side effects, PNS could be a promising option for AS treatment. Reviewing existing literature, PNS is observed to have effects in six key areas: anti-inflammation, regulation of lipid metabolism, anti-oxidative stress, inhibition of VSMCs proliferation and migration, inhibition of angiogenesis, and autophagy. This paper aims to provide a comprehensive review of the mechanisms by which PNS acts against AS, focusing on these six aspects.

### 3.1 Anti-inflammatory

AS is usually recognized as a chronic inflammatory disease, which is itself a process of inflammatory response, with a variety of inflammatory cells and inflammatory factors playing important roles in all stages of its pathogenesis (39, 40). The recruitment of leukocytes and pro-inflammatory cytokines plays a key role in the early stages of AS (41). In advanced stages of AS, inflammation also plays an important part, mainly manifested by the infiltration of large numbers of macrophages and other inflammatory cytokines into the vessel wall and the secretion of matrix metalloproteinases (MMPs), which degradation of collagen fibers in the extracellular matrix of plaques, leading to plaque rupture, bleeding and thrombosis (42).

Adhesion of monocytes to vascular endothelial cells is an essential step in the atherosclerotic process (43). Monocytes play a determining role in the expression of endothelial cell-associated adhesion molecules, which mediate the transendothelial migration of intercellular adhesion molecule-1 (ICAM-1) and vascular cell adhesion molecule-1 (VCAM-1), thereby generating an inflammatory response (44). PNS has been shown to inhibit monocyte adhesion to the endothelium and the expression of TNF-α induced endothelial adhesion molecules (such as ICAM-1 and VCAM-1), thereby inhibiting endothelial cell hyperadhesiveness (45). In addition, numerous studies have reported that the massive release of inflammatory cytokines such as TNF-α and IL-1β is a major contributor to AS. Oxidized low-density lipoprotein (ox-LDL) transiently stimulates endothelial cells, leading to an increase in the production of pro-inflammatory factors via the phosphatidylinositol-3-kinase (PI3K) and mitogen-activated protein kinase (MAPK) pathways, which is manifested by the infiltration of large numbers of macrophages and other inflammatory cytokines into the vascular wall and the secretion of MMPs that degrade collagen fibres in the extracellular matrix of the plaque, leading to plaque rupture, haemorrhage and bleeding (46). Su et al. (47) study found that PNS was able to reduce ox-LDL induced TNF-α and IL-1β expression levels in EA. hy926 cells, thus play a role in treating AS. Macrophage polarization and inflammatory responses play an important role in regulating plaque stability, and Rb1 increases IL-4 and IL-13 production and signal transducer and activator of transcription 6 (STAT6) phosphorylation and promotes anti-inflammatory M2 macrophage polarization to enhance atherosclerotic plaque stability (48).

It has been demonstrated that miR-221-3p is upregulated in AS and is involved in the pathogenesis of AS (49). Toll-like receptor-4 (NLR4) expression is upregulated in atherosclerotic plaques, and NLR4 mediates tumor nuclear factor κB (NF-κB) activation, leading to the overproduction of pro-inflammatory cytokines such as IL-1β, IL-6, and TNF-α (50). NGR1 inhibits TLR4/NF-*κ*B pathway activation by increasing miR-221 -3p expression and inhibits ox-LDL-induced apoptosis and inflammatory responses in human umbilical vein endothelial cells (HUVECs) (51). Increasing evidence suggests that activation of MAPK and nuclear NF-κB may lead to reactive oxygen species (ROS) overproduction and upregulation of VCAM-1, ICAM-1 and monocyte chemotactic protein-1 (MCP-1) (52). It has been hypothesized that excessive ROS production upregulates the expression of pro-inflammatory cytokines and adhesion molecules, thereby promoting the progression of AS (53). Dou et al. (54) found that in the aortic root of apolipoprotein e-deficient apoE-/-mice, PNS blocked ROS production, which in turn inhibited the receptor for advanced glycation end-products (RAGE)/MAPK signaling pathway, deactivated NF-κB, and reduced expression of the pro-inflammatory factors VCAM-1, ICAM-1, and MCP-1. Su et al. (47) also found that PNS can inhibit ox-LDL induced inflammatory cytokine production by activating peroxisome proliferator-activated receptor gamma (PPAR-γ), which inhibits NF-κB and MAPK activation.

### 3.2 Regulation of lipid metabolism

Disorders of lipid metabolism are independent risk factors for AS (55). Excess serum lipids penetrate the blood vessel wall and deposit in the artery lining, triggering early atherosclerotic damage (56). Cholesterol and the accumulation of cholesterol lipids are major components of atherosclerotic plaque. A large number of studies have shown that elevated levels of low-density lipoprotein cholesterol (LDL-C), triglycerides (TG), and total cholesterol (TC), as well as decreased levels of high-density lipoprotein cholesterol (HDL-C), are closely are closely linked to the onset and the development of atherosclerosis (57). Experimental studies in the AS model of apoE-/-mice showed that PNS significantly decreased TG, TC, and LDL-C levels and significantly increased HDL-C levels (58).

The ox-LDL may be involved in the progression of atherosclerotic lesions via foam cells. In the apoE-Ko AS mouse model, blood levels of ox-LDL were elevated, and PNS decreased blood levels of ox-LDL in mice (59). It is well known that low-density lipoprotein is a major causative factor in AS. The low-density lipoprotein receptor (LDLR) mediates the uptake and degradation of LDL in plasma, whereas PCSK9 binds to the extracellular structural domain of LDLR and mediates the degradation of LDLR (60, 61). 20(S)-protopanaxadiol is a class of constituents of PNS (19); it binds directly to the extracellular structural domain of the LDLR and inhibits the interaction between PCSK9 and the LDL receptor, thereby raising LDLR levels and alleviating atherosclerosis (62).

Lipid macrophages, often referred to as foam cells, are the central component of plaques and are characteristic of atherosclerotic plaques (63). Foam cells are formed from the uptake of lipids oxidised by activated monocytes/macrophages. In this process, modified lipoproteins enter the cell via a receptor and excess neutral lipids are stored as lipid droplets (64). ATP-binding cassette transporter A1 (ABCA1) reduces excess lipids in the cell, thereby preventing foam cell formation (65). PNS can reduce cholesterol ester by upregulating ABCA1 expression and lowering cholesterol ester levels, thereby inhibiting foam cell formation (66). ABCA1 and ATP-binding cassette transporter G1 (ABCG1) mediate cellular cholesterol efflux in the presence of receptors (e.g., HDL), and the liver X receptor alpha (LXRα) can directly regulate ABCA1 and ABCG1 expression (67). PNS was found to increase transcriptional activation of the LXRα gene promoter and subsequently upregulate ABCA1 and ABCG1 (36). Hypoxia-inducible factor-1α (HIF-1α) participates in atherosclerosis development by regulating glycolysis and macrophage polarization. Studies indicate that in mouse atherosclerosis models, PNS effectively suppresses abnormal glycolysis induced by HIF-1α overexpression, thereby inhibiting M1 macrophage polarization while promoting M2 macrophage polarization and modulating sphingolipid metabolism (68).

### 3.3 Anti-oxidant stress

Oxidant stress is a condition in which the body is stimulated by internal and external factors, leading to an increase in the production of ROS, resulting in an imbalance between oxidative and anti-oxidant effects in the body (69). ROS react with LDL to form ox-LDL (70); ox-LDL induces endothelial cells to express cell adhesion factors, which stimulate monocyte to macrophage differentiation and mediate lipid accumulation; it also induces the expression of CD36 and p-selectin in platelets, which promotes platelet activation and the release of chemokines, resulting in epithelial dysfunction, foam cell formation, which the promotion of atherosclerotic plaque formation (71). Numerous studies have shown that upregulation of anti-oxidant enzymes such as superoxide dismutase (SOD), reduced glutathione (GSH), and glutathione peroxidase (GPx) levels in artery wall inhibits endothelial dysfunction and ox-LDL formation (72).

Inhibition of oxidative stress by PNS is gaining widespread attention. Nuclear factor E2-related factor 2 (Nrf2) is a protein that regulates endogenous antioxidant defense and plays a crucial role in activating the downstream heme oxygenase-1 (HO-1) to counteract intracellular ROS and anti oxidative stress (73). In a mouse model of diabetic nephropathy, PNS up-regulated GSH and SOD expression and down-regulated malondialdehyde (MDA) expression by modulating the Nrf2/HO-1 pathway (74); *in vitro* experiments, NGR1 reduced the mitochondrial damage induced, limited the increase of ROS, promoted the expression of Nrf2 and HO-1, and reduced HK-2 cell apoptosis (75). PNS activated the PI3K/protein kinase (Akt)/Nrf2 pathway and up-regulates HO-1 expression, thereby anti-oxidant stress injury and ameliorating cerebral ischemia-reperfusion injury (76, 77). Zhang et al. (15) 0.2 mM H2O2 treated HUVECs for 12 h, and the cell survival rate of HUVECs was about 70%. Subsequently, HUVECs with different concentrations (20–200 mg/mL) of PNS were treated for 24 h. It was found that the cell survival rate could be increased by 37% at 200 mg/mL PNS. Compared with the control group, PNS significantly improved the stability of the cytoskeleton and maintained the normal morphology and physiological function of the cells. It has been shown that Rb1 ameliorates H2O2-induced HUVEC senescence and dysfunction through the Sirtuin-1/AMP-activated protein kinase (AMPK) pathway (78). In the rat AS model, serum NO and SOD contents were increased and TNF-α contents were reduced by PNS or Rb1 treatment. *In vitro* PNS or Rb1 treatment alleviated H2O2-mediated cytotoxicity in endothelial cells, down-regulated ox-LDL induction of p38 and VCAM-1 expression (79).

### 3.4 Inhibition of vascular smooth muscle cells proliferation and migration

VSMCs play a crucial role in the formation of AS. In the early stage, the migration and proliferation of VSMCs within the intima contribute to the formation of atherosclerotic plaques, and in the late stage, VSMCs form a fibrous cap to stabilize vulnerable plaques; also participate directly in the process of AS formation through the formation of macrophage foam cells (80). VSMCs are one of the major cellular components that maintain vascular tone and function. Studies have demonstrated that abnormal proliferation of VSMCs induces the development of AS when the endothelium is damaged.

The levels of cyclin D1 and cyclin-dependent kinase 4 (CDK4) as positive regulators of the VSMCs cycle, and P21 protein as a negative regulator of the VSMCs cycle. In one study, in VSMCs cultured after platelet-derived growth factor (PDGF) stimulation, TPNS decreased cyclin D1 and CDK4 content and increased P21 protein content, thereby inhibiting the proliferation of VSMCs (81). VSMCs were isolated from the thoracic aorta of rats and incubated with different concentrations of PNS (0.2–0.8 ug/ml), and then the cells were stimulated with PDGF for 24 h. The results showed that PNS significantly inhibited the proliferation of VSMCs by up-regulating the expression of P53, Bax, and caspase-3 proteins, and down-regulating the expression of Bcl-2 protein, induced apoptosis of VSMCs after proliferation (37). Resistin is an adipokine primarily expressed in human monocyte/macrophage lineage cells, and pathological concentrations of resistin promote VSMC proliferation and migration (82, 83). The PI3K/Akt pathway is a classical pathway for VSMCs proliferation and migration, and activation of PI3K activates phosphorylation of the downstream factor Akt to inhibit VSMCs proliferation and migration (84). Rb1 inhibits pathological concentrations of resistin that inhibition VSMC proliferation and migration, and can decrease the expression of ROS and increase the expression of SOD (85). It was shown that NGR1 inhibits VSMC proliferation and migration by inhibiting the PI3K/Akt pathway and regulating actin dynamics (86).

### 3.5 Inhibition of angiogenesis

During AS, intraplaque angiogenesis promotes AS progression and plaque destabilization. Cell adhesion molecules are highly expressed in plaque neovascularization, thereby further facilitating recruiting of inflammatory cells into the plaque (87). Vascular endothelial growth factor (VEGF) is a pro-angiogenic factor that induces VEGF expression to promote angiogenesis under hypoxic conditions. The main receptors for VEGF, VEGF-1 and VEGF-2, play crucial roles in intraplaque angiogenesis. Activation of VEGF-1 receptor results in migration of inflammatory cells and release of inflammatory factors, and activation of VEGF-2 stimulates angiogenesis, migration, and proliferation of endothelial cells (88).

It was found that PNS could alleviate atherosclerotic plaque angiogenesis and attenuate the process of AS. It was demonstrated that CD34 promotes VEGF-induced neovascularization germination and is involved in neovascularization in the retinal periphery (89). In the apoE-Ko mouse model, PNS down-regulated expressions of VEGF, CD34, and NADPH oxidase 4 (NOX4) proteins, reducing plaque area and the number of plaque neovascularization (90). In a rabbit iliac artery balloon endothelial stripping injury model, PNS downregulated the expression of VEGF and MMP-2, promoted endothelial cell regeneration, and reduced extracellular matrix thickening (91). Pigment epithelium-derived factor (PEDF) is an anti-angiogenic cytokine that inhibits VEGF-induced angiogenesis by inducing endothelial cell apoptosis and enhancing VEGF-1 cleavage (92). MiR-33a reduces cholesterol efflux and inhibits VEGF-2 signaling and angiogenesis *in vitro*. In an *in vitro* experiment, Rb1 was found to exert anti-angiogenic effects by regulating miR-33a and PEDF expression (93).

### 3.6 Regulation of autophagy

Autophagy is a highly conserved cellular degradation and recycling process in all eukaryotes. Increasing evidence suggests that autophagy plays a key role in suppressing inflammation and apoptosis, promoting efferocytosis and cholesterol efflux. Autophagy markers p62 and LC3-II are expressed in atherosclerotic plaques. Their reduction cause dead cells to accumulate in the arterial wall and contribute to plaque destabilization (94). P62 is the most prototypical substrate for selective autophagy, acting as a signaling hub that can determine cell survival by activating the TNF receptor-associated factor 6/NF-κB pathway or cell death by promoting caspase aggregation (95).

In the apoE-/-mouse AS model, Rb1 upregulates Bcl-2/Bax expression, decreases p62 expression, promotes LC3 conversion from type I to type II, and promotes autophagy to inhibit apoptosis (96). Macrophage autophagy is the most widely studied type of autophagy, and AMPK is the major kinase regulating macrophage autophagy (97); ox-LDL promotes macrophage conversion to foam cells and macrophage apoptosis promotes plaque necrosis, leading to plaque instability. Rb1 increased the expression of autophagy-related gene 5 (Atg5) and ABCA1, induced macrophage autophagy, reduced lipid accumulations in macrophage foam cells, and enhanced the stabilization of plaques (98). Evidence suggests that AMPK regulates autophagy through downstream signaling pathways, thereby inhibiting apoptosis and inflammation, promoting cholesterol efflux (99). Calcium/calmodulin-dependent protein kinase II (CaMKII) is activated in advanced atherosclerotic plaque macrophages and drives plaque necrosis by inhibiting expression of the efferocytosis receptor macrophage c-mer tyrosine kinase, whose upregulation promotes plaque stability (100). In a mouse model of acute myocardial infarction, PNS promotes autophagy in cardiomyocytes by promoting the phosphorylation of AMPK and CaMKII, upregulating LC3II/I expression and downregulating p62 expression (101). Rg1 activates the AMPK/mTOR pathway, upregulates the expression of Atg5, Beclin1, LC3, and downregulating p62 expression promotes autophagy, and inhibits apoptosis (102). Mitochondrial autophagy is an important process for the elimination of dysfunctional mitochondria regulation of multiple autophagies plays a very important role in both the early and late processes of AS (103). PNS regulates the expression of PTEN-induced putative kinase 1 (PINK1), promotes mitochondrial autophagy, reduces ROS accumulation, and also modulates calcium homeostasis dysregulation (104).

Hypoxia or ischemia induces HIF-1α activation, which maintains cell survival by activating the downstream protein BCL2 interacting protein (BNIP3), which induces mitochondrial autophagy direct binding to LC3 (105). In a rat model of myocardial ischemia-reperfusion injury, PNS upregulated the expression of HIF-1α, BNIP3, Atg5, LC3, and Beclin-1 in myocardial tissues, which demonstrated that PNS enhances mitochondrial autophagy via the HIF-1*α*/BNIP3 pathway (106).

## 4 Dose-response relationship and clinical translation considerations

As shown in Table 1, the optimal concentration of Panax notoginsenosides *in vitro* cell models is typically around 20 μM, while in AS animal models, the optimal oral dosage generally ranges from 60 to 120 mg/kg/day. Different monomers exhibit varying concentrations required to act on distinct pathways, demonstrating target specificity. Converting animal doses to human equivalent doses is a critical step in preclinical research. Taking the effective dose of 60 mg/kg/d for PNS in mouse studies as an example, preliminary estimation based on body surface area conversion suggests a rough human equivalent dose of approximately 6.6 mg/kg/d. Current dose calculations remain theoretical projections; actual clinical dosages must be determined through rigorous clinical trials. Current research still faces significant limitations: First, most studies utilize PNS extracts with inconsistent ratios of individual components, making precise dose-response relationships difficult to define. Second, research on PNS's oral bioavailability, tissue distribution, and metabolic conversion remains insufficient, complicating the correlation between effective *in vitro* concentrations and required *in vivo* dosages.

**Table 1:** PNS dosage schedule for AS treatment.

Compounds/extracts	Model	Target/mechanism	Optimal dose	Administration time	References
PNS	ApoE-/- mouse	Modulation of inflammatory responses and lipid regulation	12.0 mg/Kg/d	8 weeks	(45)
PNS	HCAECs	Modulation of inflammatory responses and lipid regulation	300 μg/ml	24 h	(45)
NGR1	EA.hy926 cell	Activates PPAR*γ* expression and inhibits NF-*κ*B and MAPK activation	100 μM	24 h	(47)
Rb1	Macrophage	Macrophage polarization	20 μM	24 h	(48)
PNS	ApoE-/- mouse	Inhibits RAGE/MAPK signaling pathways	60 mg/Kg/d	4 weeks	(54)
PNS	ApoE-/- mouse	Inhibits NF-κB signaling pathway	180 mg/Kg/d	8 weeks	(58)
PNS	ApoE-KO mouse	lipid regulation	60 mg/Kg/d	12 weeks	(59)
PPD	HepG2 cells	Inhibit LDLR degradation	10 μM	24 h	(62)
PPD	ApoE-KO mouse	Inhibit LDLR degradation	60 mg/Kg/d	12 weeks	(62)
PNS	Macrophage	ABCA 1, LXR1	80 mg/L	24 h	(66)
PNS	ApoE-/- mouse	HIF-1*α*	120 mg/Kg/d	4 weeks	(36)
PNS	RAW264.7 cells	HIF-1α	150 μg/mL	24 h	(36)
PNS	Microvascular endothelial cells of the brain	Activates PI3K/Akt/Nrf2			
Antioxidant Signaling Pathway	400μg/mL	24h	78		
PNS	Mouse microvascular cerebral endothelial cells	NrF2, NF-κB	400 μg/mL	24 h	(77)
Rb1	HCAECs	Activates SIRT1/AMPK pathway	20 μM	24 h	(78)
PNS	HCAECs	Inhibits NrF2, p38 -VCAM-1 Signal Pathway	100 μg/ml	24 h	(79)
PNS	VSMCs	p53, Bax, caspase-3 and Bcl-2	800 μg/mL	24 h	(37)
Rb1	HCAECs	ROS, SOD	20 μM	24 h	(85)
NGR1	HCASMCs	PI3K/Akt signaling	10 μM	24 h	(86)
PNS	ApoE-KO mouse	NOX4, VEGF	60 mg/Kg/d	12 weeks	(90)
Rb1	HCASMCs	miR-33 a/PPAR-γ	20 μM	24 h	(93)
Rb1	ApoE-/- mice	Inflammation, lipid metabolism, regulation of autophagy	10 mg/Kg/d	8 weeks	(96)
Rg1	Raw264.7 macrophages	AMPK/mTOR signaling pathway	50 μM	48 h	(102)

## 5 Conclusions and future perspectives

**Figure 2:**
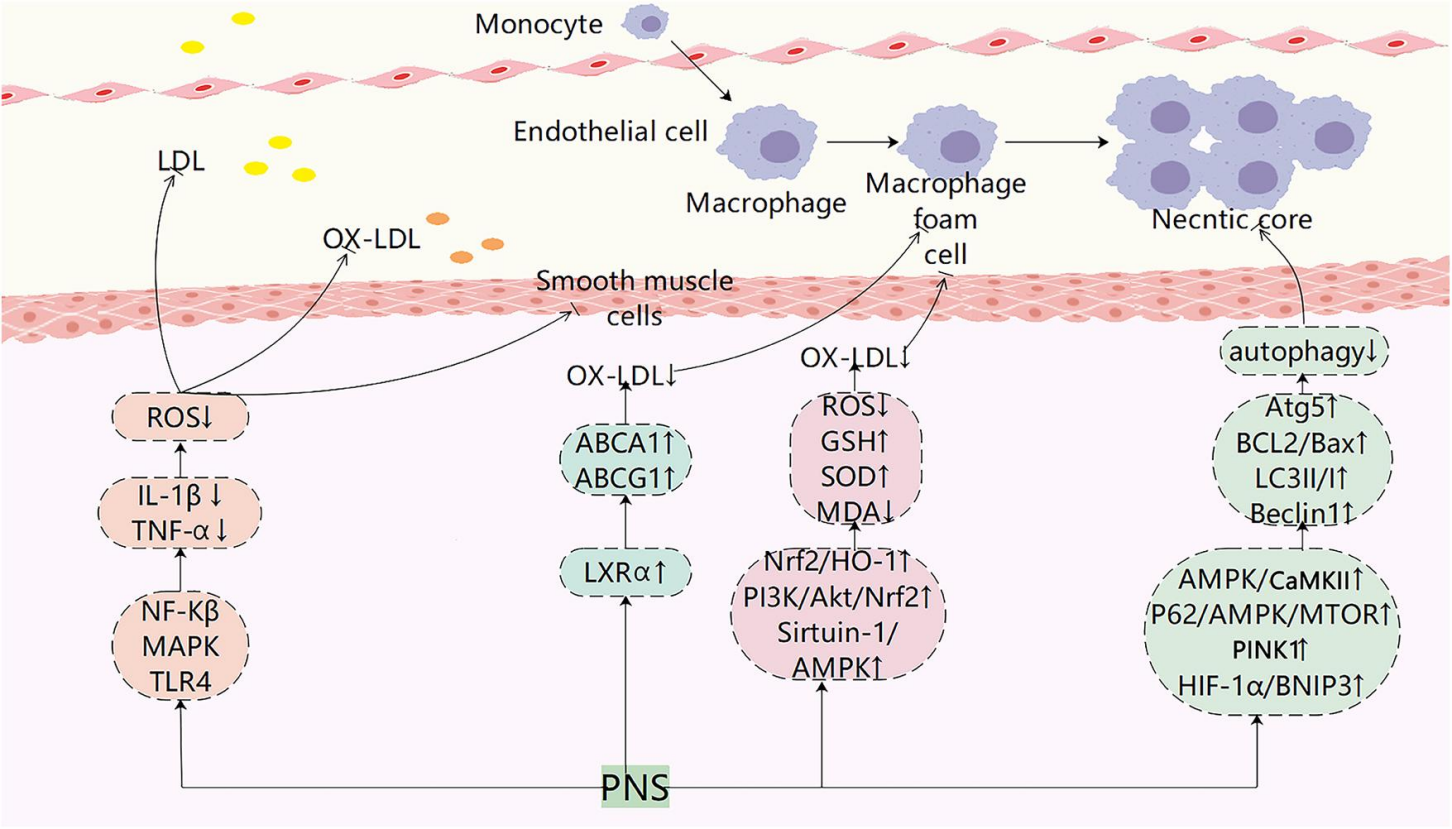
Pharmacological mechanisms of PNS in atherosclerotic plaque evolution. PNS exerts anti-atherosclerotic effects by modulating multiple signaling pathways, including suppressing inflammatory responses, regulating lipid metabolism, reducing LDL oxidation, inhibiting foam cell formation, suppressing smooth muscle cell migration, and regulating autophagy.

This research provides strong evidence supporting the use of PNS in the clinical treatment of AS. Recent studies have shown significant progress in the foundational understanding of AS with PNS interventions (Figure 2; Table 2). PNS has been found to modulate key pathways such as PI3K, MAPK, and TLR4/NF-κB, leading to reduced expression of inflammatory factors like TNF-α and IL-1β, as well as decreased cholesterol deposition. Additionally, PNS blocks pathways like Nrf2/HO-1, PI3K/Akt/Nrf2, AMPK, and HIF-1α/BNIP3, resulting in lower ROS levels, enhanced antioxidant defenses, protection of VSMCs and endothelial cells, and promotion of autophagy. AS emerging research areas such as exosomes, intestinal flora, and ferroptosis gain traction in AS studies, further investigations are warranted to explore the potential of PNS in regulating these mechanisms for AS treatment. Compared to other plant saponins (such as ginsenoside Rc) or phenolic compounds (such as curcumin), PNS demonstrates unique potential for multi-pathway synergistic effects in combating AS. However, the clinical application of PNS still faces significant challenges: First, its low oral bioavailability, primarily related to intestinal metabolism and permeability; second, the complex composition of PNS, where the specific monomer or combination playing a key role and its definitive target proteins remain to be confirmed; third, existing studies are predominantly preclinical models, lacking high-quality human evidence-based medical data. To address these limitations, future research should focus on: enhancing bioavailability through structural modifications or novel delivery systems; precisely identifying molecular targets using chemical biology approaches; and conducting rigorously designed randomized controlled clinical trials to provide novel perspectives and insights.

**Table 2:** Mechanism of action of PNS in aS.

Chemicals	Effect	Mechanisms	References
PNS	Anti-inflammatory	Inhibition of adhesion factor expression and lipid lowering	(45)
NGR1		Activates PPARγ expression and inhibits NF-κB and MAPK activation	(47)
Rb1		Promotes anti-inflammatory M2 macrophage polarization	(48)
NGR1		Inhibition of TLR4/NF-κB pathway activation	(51)
PNS		Reduced RAGE/MAPK pathway expression and inhibited NF-κB activation	(54)
PNS	Regulation of lipid metabolism	inhibited NF-κB activation, lipid lowering	(58)
PNS		Reduction of lipid levels and down-regulation of CD40 and MMP-9 expression	(59)
PNS		Upregulation of LXRα, ABCA1 and ABCG1 expression	(36, 66)
PNS, NGR1	Anti-oxidant stress	Upregulation of the Nrf2/HO-1 pathway against oxidative stress	(74, 75)
PNS		Activation of PI3K/Akt/Nrf2 pathway and upregulation of HO-1 expression against oxidative stress injury	(76, 77)
PNS		Improve cell viability and maintain cytoskeleton stability	(15)
Rb1		Activation of Sirtuin-1/AMPK pathway against cellular senescence	(78)
PNS, Rb1		Inhibition of ROS/TNF-α/p38/VCAM-1 pathway inhibits monocyte adhesion	(79)
PNS	Inhibition of VSMC proliferation and migration	Inhibition of ERK pathway activation to suppress VSMC proliferation	(81)
PNS		Up-regulation of p53, Bax, and caspase-3 expression and down-regulation of Bcl-2 expression inhibited proliferation and induced apoptosis in VSMCs	(37)
Rb1		Inhibition of VSMC proliferation and migration	(85)
NGR1		Inhibition of PI3K/Akt pathway activation suppresses VSMCs proliferation	(86)
PNS	Inhibition of angiogenesis	Downregulation of VEGF and NOX4 expression reduces plaque angiogenesis	(90)
PNS		Promote endothelial regeneration and reduce endothelial thickening	(91)
Rb1		Regulation of miR-33a and PEDF expression exerts anti-angiogenic effects through PPAR-γ signaling pathway	(93)
Rb1	Regulation of autophagy	Regulation of BCL-2 family-associated apoptosis to promote autophagy	(96)
Rb1		Increases AMPK phosphorylation and induces macrophage autophagy	(98)
PNS		Activation of the AMPK/CaMKII pathway promotes autophagy	(101)
Rb1		Activation of AMPK/mTOR pathway promotes macrophage autophagy	(102)
PNS		Induction of PINK1 expression regulates autophagy	(104)
PNS		Activation of the HIF-1α/BNIP3 pathway enhances mitochondrial autophagy in myocardial tissue	(106)
